# Basaloid Squamous Cell Carcinoma, an Aggressive and Rare Cancer of the Oral Cavity: Can We Prevent It at the Primary Care Setting?

**DOI:** 10.7759/cureus.28775

**Published:** 2022-09-04

**Authors:** Beatriz M Cunha, Marisa Sousa, Hugo B Sousa

**Affiliations:** 1 Family Medicine, Unidade de Saúde Familiar de Samora Correia (USF Samora Correia) ACeS Estuário do Tejo, ARSLVT, Samora Correia, PRT

**Keywords:** oral cancer screening, primary care, prevention, risk factors, oral cancer, basaloid squamous cell carcinoma

## Abstract

Oral cavity cancer represents about 2%-3% of all cancers worldwide, with more than 355,000 new cases per year, one-third of which are reported in developed countries. Oral cancer is also known to be extremely aggressive when detected late, thus presenting one of the lowest cancer survival rates. It is estimated that as much as 90% of oral cancers are attributable to tobacco and/or alcohol consumption and that high-risk human papillomaviruses (HPV) infections pose an independently increased risk for their development. Therefore, it can be a preventable disease when associated with changes in lifestyle and possible modifiable risk factors, combined with early and preventive intervention.

Basaloid squamous cell carcinoma (BSCC) constitutes an aggressive and rare form of oral cancer, being one of the rarest and most aggressive variants of squamous cell carcinoma (SCC, the most common), and usually presents as a high-grade disease with a poor prognosis. It is typically associated with heavy smoking and alcohol abuse, occurring most commonly in older men.

Here, we report a clinical case of a 60-year-old man with excessive consumption of both tobacco and alcohol, poor oral hygiene, and partial edentulousness who came to our primary health department with complaints of odynophagia twice in a four-year time-lapse. The first time, two whitish ulcerated lesions on the left tonsil were detected and biopsied but revealed a negative histological result. After four years, he came again to our primary health care department with similar complaints of odynophagia and also sore throat with radiation to the right ear, accompanied by globus sensation and anorexia. No suspicious lesions were detected, except a globally hyperemic oropharynx. Considering the history of abusive consumption, no improvement with symptomatic treatment, and persistent clinical signs, an extended diagnostic approach was carried out. After four months, a pharyngeal mass measuring 53 mm was detected on pharyngeal-neck computed tomography (CT), and the diagnosis of a BSCC located in the right tonsillar pillar and base of the tongue was finally determined.

Unlike other cancers that have been detected earlier through screening programs, oral cancer is often detected at an advanced stage, compromising survival and quality of life. The opportunity to intervene early and preventively in consumption habits, promote healthy lifestyles, and try to prevent disease is unique at the primary care level. Moreover, opportunistic screening through a thorough examination of the oral cavity is extremely important for timely diagnosis and treatment.

## Introduction

The increasing prevalence of non-infectious diseases, including oncological diseases, has led to a growing concern to create urgent and effective public health responses. Oral cancer (i.e., cancer of the oral cavity, oropharynx, and lips) represents about 2%-3% of all cancers worldwide and constitutes a preventable disease when associated with risk factors and modifiable lifestyles. Known for its aggressiveness when detected late, oral cancer had 2020 an estimated annual incidence of 355,000 new cases, one-third of which were in developed countries and about 50% in advanced stages of the disease [1].

The rates for men are currently twice as high as for women, and approximately 95% of carcinomas occur in persons over 45 years old, with an average age at the time of diagnosis of 60 years; however, there is an increasing trend to affect younger men and women [2].

More than 90% of all oral cancers are squamous cell carcinomas (SCC), being basaloid squamous cell carcinoma (BSCC) one of the rarest (0,6-0,8% of all SCC) and aggressive variant, usually detected at an advanced stage already with liver and/or lung metastases [3-5]. According to a total of 231 published BSCC cases, this variant has a predilection for the floor of the mouth (42,8%), tongue (19,1%), retromolar trigone (12,5%), and alveolar ridge/gingiva (11,8%), followed by the palate (6,6%) and buccal mucosa (5,9%), being rare in the tonsillar pillar (1,3%) [4-5]. In the 31-year retrospective study by Schuch and collaborators [5], the clinical presentation of BSCC was usually an asymptomatic ulcer with a mean evolution time of 6,8 months (range 3-12 months).

It is estimated that as much as 90% of cancers of the oral cavity worldwide, such as BSCC, are attributable to tobacco use, alcohol consumption, or a combination of both, with the latter posing a much greater risk than the use of either substance alone [6-7]. Human papillomavirus (HPV), HPV-p16 in particular, has been implicated as an etiological agent for the development of a subset of SCC, especially at the base of the tongue and tonsils in younger individuals, compared to the HPV-negative types that show a worse prognosis [8-9]. Besides alcohol, tobacco, and HPV infection, poor oral hygiene, poor diet (vitamins A and C deficits; diet lacking vegetables and fruits), exposure to UV light (particularly for labial cancer), and an immunosuppression state (especially, HIV-related) also pose as risk factors for oral cancer development [10-11].

Despite the easy accessibility of the oral cavity (only comparable to the skin) for direct examination, these malignant tumors are not usually detected until an advanced stage of their development, and thus, the survival rate for oral cancer has remained virtually unchanged and low in the last four decades [1-2,6]. The key to better outcomes regarding oral cancer is thus timely diagnosis and treatment, that is, early intervention with screening through routine oral medical examination and oral health education and training programs.

Here, we present the clinical case of a 60-year-old man with heavy habits of tobacco and alcohol consumption, on which we have diagnosed a BSSC located at its right tonsillar pillar and base of the tongue after extensive investigation. Initially, he only presented subtle symptoms without suspicious lesions, but in four months, it has evolved into an advanced disease.

This article was previously presented as a meeting abstract and e-poster at the 39^th^ Meeting of the “Associação Portuguesa de Medicina Geral e Familiar” (APMGF) on March 31^st^, 2022 (Aveiro, Portugal).

## Case presentation

We report a clinical case of a 60-year-old man, followed by our primary health care department initially in a phase of severe social poverty, unemployed and without a support network, with excessive consumption of tobacco (42 smoking pack-years) and alcohol (441 g per week), poor oral hygiene and partial edentulousness.

In 2017, he complained of intermittent odynophagia, and two whitish ulcerated lesions on the left tonsil were detected, with a negative histological result. With intervention in a primary care setting, which took place between 2017 and 2020, he abandoned alcohol, reduced tobacco consumption, and entered a job. He did not report any other oral cavity-related symptoms during that time.

In 2021, he reported similar complaints of odynophagia and sore throat with radiation to the right ear, accompanied by a globus sensation and anorexia. The patient did not have constitutional symptoms or any other complaints. The otoscopy was unaltered, and the oropharynx was globally hyperemic, with no suspicious lesions. No limited movement of the tongue or limited ability to open the mouth was detected. No lymph nodes were found to be enlarged on cervical palpation. The patient was medicated symptomatically without alleviation of complaints. Blood analysis and ultrasonography of the neck showed no abnormal findings in our first approach.

**Figure 1:**
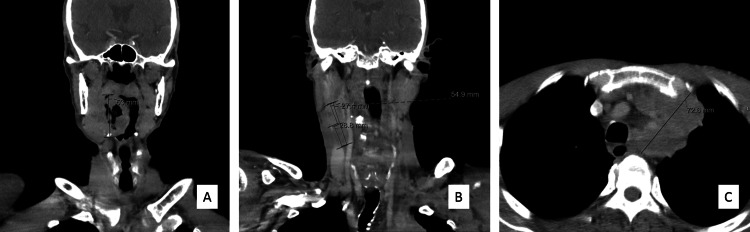
Pharyngeal-neck and thorax computed tomography scans (A) Coronal view of contrast-enhanced pharyngeal-neck computed tomography (CT) scan showing an expansive and infiltrative lesion on the right lateral wall of the oropharynx that extends from the plane of the soft palate to the base of the tongue and pharyngoepiglottic fold, reaching a maximum longitudinal axis of 53 mm (ruler). The lesion invades the root of the tongue and the sublingual space; (B) Coronal view of contrast-enhanced pharyngeal-neck CT revealing two coalescing adenopathies on the right involving levels II and III and reaching 55 mm (ruler) in the longest longitudinal axis - the uppermost solid and measuring 27 mm (ruler) and the lower, predominantly necrotic, measuring 29 mm (ruler) in the longest axis; and (C) Axial view of contrast-enhanced staging thorax CT revealing mediastinal involvement with heterogeneous adenopathies containing central areas of necrosis - the most expressive conglomerate surrounds the supra-aortic trunks, without a cleavage plane with the aortic arch, with a 73 mm longest axis (ruler) and causing a deviation of the trachea to the right

Taking into account the history of tobacco and alcohol abusive consumption and persistent clinical signs that progress to pain on mastication and swallowing and later to dysphagia, an extended diagnostic approach was carried out. The patient underwent upper endoscopy that revealed an erosive gastropathy that was *Helicobacter pylori *positive; chest X-ray and abdominal ultrasonography showed no relevant abnormal findings. Finally, after four months of investigation, a parapharyngeal mass located in the right tonsillar pillar and base of the tongue measuring 53 mm, plus jugulo-carotid adenopathies, were detected on pharyngeal-neck Computed Tomography (CT) (Figure 1A and Figure 1B). At this stage, a conglomerate of right cervical adenopathies measuring approximately 2 per 4 cm, poorly mobile, were also found on patient examination.

The patient was referred to the otorhinolaryngology and oncology departments, and through nasofibroscopy, the mass located at the right lateral wall of the oropharynx was identified and submitted for biopsy. Cancer staging through a series of CT scans revealed a cT4aN2cM1 tumor not only with cervical lymphadenopathies but also with extensive mediastinal lymphadenopathies (the most expressive conglomerate with 73 mm, Figure 1C) and a pulmonary nodule with 15 mm. Histological diagnosis was of a BSCC, HPV-p16 negative, with poor prognosis. The patient is currently undergoing palliative chemotherapy and institutionalized in the nursing home where he was working, thus having daily support.

## Discussion

In this case, after intervention in terms of heavy consumption habits (alcohol and tobacco) and social disintegration, it was possible to improve the quality of life of our patient and reintegrate him socially and into work. Despite the gain in the reduction of risk factors, we witnessed the appearance, within four months, of a clinical picture with mild complaints but with a rare and very aggressive final diagnosis. This time-lapse for BSCC is in accordance with previous studies, as determined by Schuch and collaborators [5], that report a range evolution time of 3 to 12 months. Moreover, it is also described that the risk of tobacco-related cancers of the upper aero-digestive tracts declines only five years after smoking cessation and may approach that of nonsmokers after 10 years of abstention [12]. On the other hand, the risk reduction of head and neck cancer after alcohol abstention reaches the level of never drinkers only after 20 years [13].

The potentially malignant lesions that may precede oral cavity cancer, including BSCC, are usually asymptomatic and subtle, as is cancer itself in its early stages, which further contributes to late diagnosis [1,5,10]. On the other hand, symptoms like discomfort, dysphagia, sore throat, odynophagia, limited movement of the tongue, limited ability to open the mouth, cervical and submandibular nodes, weight loss, and loss of sensory function are associated with advanced stages of oral cancer [1,7,10]. In this particular case, our patient came to our primary health care department for the first time with subtle symptoms and suspicious lesions on the throat, but we rapidly excluded a diagnosis of malignancy by biopsy of the lesions. After four years, this patient did not present any premalignant lesion on the oral cavity, an uncommon case, and was seeking our help with subtle symptoms that have quickly evolved into symptoms of advanced disease at the time of diagnostic imaging.

Unlike other cancers that have been detected earlier through screening programs, oral cancer continues to be detected at an advanced stage (50% are diagnosed at stages III or IV), compromising the quality of life and survival [1-2,4,6]. Despite all the advances in treatment (surgery, radiotherapy, and chemotherapy), the five-year survival rate for oral cavity cancer remains at about 55% [10]. Therefore, it is essential to intervene early and preventively in consumption habits (for example, by referring patients to alcohol and/or smoking cessation consultations), promoting healthy lifestyles, and carrying out timely opportunistic screening for oral cancer to reduce its morbidity and mortality.

Up to 70% of oral cancers are preceded by premalignant oral lesions, such as persistent red or white patches (erythroplakia and leukoplakia, respectively), ulcerations, indurations, bleeding, or nodes in the mouth, accessible to visual inspection [14]. Moreover, it has been shown that screening high-risk groups by systematic visual oral examinations are cost-effective and feasible [2,15-16].

Opportunistic oral cancer screening examinations are generally conducted by oral health practitioners, but they could also be implemented in the primary care setting as an important means for early identification and diagnosis, especially in places where health care resources are low [2,15-17]. Primary care providers should question their patients about risk factors, particularly tobacco and alcohol consumption, at each visit, and supplement this information with individual observation [18-19]. According to the National Institute of Health [18] and the World Health Organization [19], a thorough visual inspection and palpation of oral soft tissues (tongue, floor of the mouth), extra-oral regions of the head and neck (temporal, masseter and mandibular muscles, temporomandibular joints, parotid, and submaxillary glands) and regional lymph nodes should be an integral part of the routine physical examination of all patients. Any abnormality that lasts for more than three weeks or a suspicious lesion should be re-evaluated and referred for biopsy [15,17]. Those aged 40 years or older are particularly important, especially if they are male, smokers, and excessive alcohol users [6-7,18-19]. People with a history of past oral cancer, HPV infection, immunodeficiency, and UV exposure are also at greater risk for oral cancer development [8-11,18-19]. Therefore, opportunistic screening is advised for those who meet the conditions described above [16,18-19]. Patient health education should be a key element in the approach of such patients, promoting healthy lifestyles and avoiding risky behaviors, given that almost 90% of oral cancers are caused by tobacco and/or alcohol use. Patients should be informed about the association between tobacco, alcohol, and oral cancer [18-19].

## Conclusions

In the last decades, there has been a huge advance in the early detection of several malignant neoplasms leading to a better prognosis; however, this did not happen with cancer of the oral cavity. The survival rate is directly related to the stage of the disease at the time of detection. Thus, the efforts of health professionals, especially in the primary care setting, to prevent the disease by promoting healthy lifestyles and making an early diagnosis aim not only to reduce its incidence but also to improve the life expectancy of those patients.

Early-stage cancers are often asymptomatic and mimic benign conditions, reducing the likelihood for the patients to seek health care. Therefore, screening provides an opportunity for early detection in the population with important risk factors. Clinicians must become increasingly able to routinely identify pre-malignant and malignant lesions, the latter in the early stages of their development since intervention at this stage is more effective. Only an early diagnosis makes it possible to offer better treatment to those patients, improving prognosis and survival, with a great impact on the patient's quality of life.
